# Disparities in Clinical and Experimental Pain Between Non-Hispanic White and Asian American Individuals With Knee Osteoarthritis and the Role of Pain Catastrophizing: Pilot Study in Florida

**DOI:** 10.2196/64415

**Published:** 2025-02-25

**Authors:** Chiyoung Lee, C. Kent Kwoh, Juyoung Park, Lindsey Park, Hyochol Ahn

**Affiliations:** 1 College of Nursing University of Arizona Tucson, AZ United States; 2 The University of Arizona Arthritis Center Tucson, AZ United States; 3 Division of Rheumatology College of Medicine University of Arizona Tucson, AZ United States

**Keywords:** Asian American, non-Hispanic White, osteoarthritis, pain, pain catastrophizing

## Abstract

**Background:**

Although a few studies have delineated the disparities in knee osteoarthritis (KOA) pain between non-Hispanic White and Asian American individuals, a significant research gap persists in elucidating the mechanisms underlying these differences.

**Objective:**

This pilot study aims to examine psychological factors, specifically pain catastrophizing and negative affect, as potential explanatory mechanisms for these dissimilarities.

**Methods:**

A cross-sectional design was used. Forty community-dwelling participants aged 50-70 years with self-reported KOA pain, including 20 non-Hispanic White and 20 Asian American individuals, were recruited in North Central Florida. Clinical KOA pain intensity was assessed using the Western Ontario and McMaster Universities Osteoarthritis Index (WOMAC) and the 4 subscales of the Short-Form McGill Pain Questionnaire-2. Quantitative sensory testing was conducted to measure experimental sensitivity to heat- and mechanically induced pain, including heat pain, pressure pain threshold, and punctate mechanical pain, as well as inhibitory pain processes through conditioned pain modulation. Pain catastrophizing was evaluated using the Coping Strategies Questionnaire-Revised Pain Catastrophizing subscale, while negative affect was assessed using the Positive and Negative Affect Schedule. Bayesian mediation analyses were used to examine both direct and indirect effects (mediation) between variables.

**Results:**

Asian American individuals exhibited higher pain catastrophizing scores than non-Hispanic White individuals. Pain catastrophizing, at high levels, contributed to WOMAC and Short-Form McGill Pain Questionnaire-2, which measured clinical pain. Race had no direct effects on these pain scores but exerted significant indirect effects via pain catastrophizing (WOMAC pain: 0.96, 95% CI 0.03-2.16; continuous pain: 0.84, 95% CI 0.18-1.70; intermittent pain: 0.78, 95% CI 0.03-1.71; neuropathic pain: 0.43, 95% CI 0.03-0.95; and affective pain: 1.05, 95% CI 0.24-1.99); thus, pain catastrophizing likely fully mediated the relationship between race and these pain measures. While Asian American individuals reported greater experimental pain sensitivity (heat pain, pressure pain threshold, and punctate mechanical pain) than non-Hispanic White individuals, these racial effects were not mediated by pain catastrophizing. Asian American individuals reported higher negative affect scores compared with non-Hispanic White individuals; however, negative affect did not mediate the relationship between race and any pain measures.

**Conclusions:**

The results demonstrate the contribution of pain catastrophizing to clinical pain in Asian American individuals with KOA and identify it as a potential mechanism underlying group differences in KOA pain between non-Hispanic White and Asian American individuals. However, caution is warranted due to the exploratory nature of this study and the treatment of Asian American individuals as a monolithic sample. Hence, future replication with larger and more diverse samples is necessary. Additionally, the lack of mediation effects of pain catastrophizing in the relationship between race and experimental pain suggests the need to explore other factors, such as biological, genetic, social, and environmental influences. Moreover, further research is essential to clarify the role of negative affect.

## Introduction

### Background

Symptomatic knee osteoarthritis (KOA), characterized by pain, disability, and diminished quality of life, is a prevalent joint disorder in middle-aged and older adults [1]. Traditional perspectives have primarily linked KOA pain to structural joint changes; however, the weak correlation between radiographic findings and clinical symptoms suggests that structural abnormalities alone do not comprehensively account for the pain experienced by sufferers [2-5]. This indicates that additional factors, including psychosocial influences, may significantly impact the severity and perception of OA-related pain [6].

The prevalence of symptomatic KOA is projected to increase in racially and ethnically marginalized groups [7]. Although significant disparities in clinical and experimental pain have been reported across racial and ethnic groups with KOA, relevant studies have predominantly compared non-Hispanic White with African American individuals [8]. Asian American individuals, a rapidly growing minority, have been underrepresented in pain research despite emerging evidence indicating that they experience greater KOA-related pain than non-Hispanic White individuals [9], challenging cultural stereotypes of stoicism. To date, no studies have delineated the mechanisms underlying the differences between non-Hispanic White and Asian American individuals.

The biopsychosocial model of pain recognizes the importance of various psychological factors in pain [10]. One such factor, pain catastrophizing—cognitive and affective pain appraisal characterized by the tendency to address and magnify the threat value of painful stimuli and feel helpless owing to pain—is reportedly significantly correlated with increased knee pain severity in both clinical and experimental settings [11-13]. Notably, Asian American individuals have been reported to exhibit higher levels of pain catastrophizing, possibly influenced by acculturative stress and cultural practices affecting pain perception and response [14]. The greater use of pain catastrophizing among racial and ethnic minorities can also be attributed to health disparities resulting from structural and systemic barriers to adequate pain treatment and biased health care interactions [15,16], possibly promoting negative perceptions regarding pain management and the belief that their pain cannot be controlled and is likely to worsen. Considering the association between higher levels of pain catastrophizing and increased KOA pain, pain catastrophizing plausibly mediates the racial disparities observed in KOA pain between non-Hispanic White and Asian American individuals.

In addition to specific pain-related factors, broader elements, such as depression and negative affect, influence knee pain experiences, and this prevails in situations involving clinical and experimentally induced pain [17-19]. Ahn et al [20] found higher levels of depression to occur in Asian American individuals with self-reported KOA pain compared with those in age- and sex-matched non-Hispanic White individuals, and such variations in depression evidently mediated racial group differences in clinical and experimental pain. Negative affect, a general predisposition to experiencing aversive mood states, has been associated with racial discrimination and psychological distress among Asian American individuals [21]. Daily microaggressions contribute to higher levels of mental health symptoms and negative affect among Asian American individuals [22-24] and specific Asian American groups [25]. To date, the role of negative affect in racial disparities in KOA pain between non-Hispanic White and Asian American individuals has not yet been investigated.

### Objectives

This pilot study aims to investigate psychological factors, specifically pain catastrophizing and negative affect, as potential explanatory mechanisms underlying group differences in KOA pain between non-Hispanic White and Asian American individuals. Elucidating these mechanisms may inform the development of targeted interventions that improve KOA pain management in the understudied Asian American population and help address pain disparities across racial groups.

## Methods

### Study Participants

This cross-sectional analysis used baseline data from the randomized controlled trial registered at ClinicalTrials.gov (NCT02512393) to examine the efficacy of transcranial direct current stimulation on KOA pain. Detailed selection criteria and enrollment procedures have been documented previously [26]. In summary, at baseline, 40 participants with KOA pain (20 non-Hispanic White and 20 Asian American individuals) were recruited in North Central Florida between September 2015 and August 2016 through local advertisements. Participants were eligible if they were aged 50-70 years, had self-reported unilateral or bilateral KOA pain as per American College of Rheumatology criteria, could speak and read English, and were willing and able to provide written informed consent before enrollment. In our sample of Asian American individuals, detailed information on subgroup ethnicities, languages, cultural backgrounds, and demographic characteristics was not gathered during data collection and is therefore unavailable in this study.

Exclusion criteria ensured participants did not have concurrent medical conditions that could confound osteoarthritis-related outcomes or coexisting diseases that could impede protocol completion, including (1) prosthetic knee replacement or nonarthroscopic surgery on the affected knee; (2) serious medical illness, such as uncontrolled hypertension, heart failure, or recent history of acute myocardial infarction; (3) peripheral neuropathy; (4) systemic rheumatic disorders, such as rheumatoid arthritis, systemic lupus erythematosus, and fibromyalgia; (5) alcohol or substance abuse; (6) cognitive impairment (ie, Mini-Mental Status Examination score≤23); (7) history of brain surgery, tumor, seizure, stroke, or intracranial metal implantation; (8) pregnancy or lactation; and (9) hospitalization for psychiatric illness within the past year.

### Measurement

The collected basic characteristics included age, sex, BMI (kg/m²), Kellgren-Lawrence radiographic grade, employment status, marital status, educational attainment, and household income.

### Clinical KOA Pain

#### The Western Ontario and McMaster Universities Osteoarthritis Index Pain Subscale.

Average knee pain for the past 48 hours was measured by the pain subscale of the Western Ontario and McMaster Universities Osteoarthritis Index (WOMAC), which consisted of 5 items on a 0-4 Likert scale measuring the pain severity during walking, climbing stairs, sleeping, resting, and standing [27]. The participants’ responses to each pain question were summed up to derive an aggregated score for pain intensity (range: 0-20). The subscales in WOMAC demonstrate reliability and validity in evaluating patients with KOA [28,29].

#### Short Form McGill Pain Questionnaire-2

The Short Form McGill Pain Questionnaire-2 (SF-MPQ-2) has been validated and widely used to assess the multidimensional qualities of pain [30]. It consists of 4 subscales, including continuous pain (6 items: throbbing pain, cramping pain, gnawing pain, aching pain, heavy pain, and tender), intermittent pain (6 items: shooting pain, stabbing pain, splitting pain, electric-shock pain, and piercing), neuropathic pain (6 items: hot-burning pain, cold-freezing pain, pain caused by light touch, itching, tingling or pins and needles, and numbness), and affective description of pain (4 items: tiring-exhausting, sickening, fearful, and punishing-cruel). Each subscale score was computed as the average of answered items, with higher scores indicating greater pain intensity.

#### Quantitative Sensory Testing

A multimodal Quantitative Sensory Testing (QST) battery was used to assess pain sensitivity using precisely controlled protocols that elicit pain with thermal and mechanical stimuli, as well as inhibitory pain processes. This includes heat pain (ie, threshold and tolerance), pressure pain threshold (PPT), punctate mechanical pain (PMP), and conditioned pain modulation (CPM). The sequence of heat and mechanical testing was randomized and counterbalanced, while CPM was always administered last to minimize any potential carryover effects. The same researcher performed QST on each participant throughout the study, and all participants were provided with standardized recorded instructions to prevent bias during data collection and to enhance the reliability of the results.

#### Thermal Testing Procedures

All thermal stimuli were delivered using a computer-controlled TSA-II NeuroSensory Analyzer (Medoc Ltd) to measure heat pain thresholds and heat pain tolerances on both the index knee and the ipsilateral ventral forearm using an ascending method of limits. At each body site, the thermode position was moved between trials to prevent sensitization or habituation of cutaneous receptors. Starting from a baseline of 32 °C, the thermode temperature increased at a rate of 0.5 °C per second until participants responded by pressing a button on a handheld device. Participants were instructed to press the button when heat first becomes painful to assess the heat pain threshold, and when they could no longer tolerate the heat pain to assess heat pain tolerance. Three trials of heat pain threshold were conducted at the first test site, followed by 3 trials of heat pain tolerance were conducted. Then, 3 trials each at the second test site were conducted, with a 5-minute rest period between sites. The average of the 3 trials was calculated for each individual, providing overall heat pain threshold and tolerance temperatures for analysis.

#### Mechanical Testing Procedures

Mechanical pain response was measured via 2 approaches. First, PPT was assessed by applying blunt mechanical pressure to deep tissues (ie, muscle and joint) via a handheld digital pressure algometer (Wagner). Increasing pressure was applied at a constant rate of 0.3 kgf/cm^2^ per second to measure the PPT at 4 sites––the medial and lateral aspects of the index knee, ipsilateral quadriceps, and trapezius. The order of testing sites was counterbalanced and randomized. For assessing PPT, participants were instructed to inform the experimenter when the sensation “first becomes painful” occurred, and the pressure was recorded. The results of the 3 trials at each body site were averaged for each site, and then these PPTs at 4 sites were averaged to derive an overall measure of PPT. Second, PMP stimuli evaluated cutaneous mechanical sensitivity on both the index patella and the back of the ipsilateral hand. We used calibrated nylon monofilament that delivered a target force of 300 g to obtain verbal ratings of the pain intensity on a scale of 0 (no pain sensation) to 100 (the most intense pain sensation imaginable) following 10 contacts at the rate of 1 contact per second. An overall score for each site was computed by averaging across 2 trials.

#### Conditioned Pain Modulation

Ten minutes after assessing the thermal or mechanical pain, the CPM was evaluated. CPM reflects the endogenous pain inhibitory pathway (ie, descending pain inhibition) also known as the “pain inhibits pain” paradox [31]. CPM was assessed by determining the change in PPT on the trapezius, immediately following the immersion of the contralateral hand up to the wrist in the cold-water bath (12 °C) for 1 minute. The initial preimmersion PPT measurement was conducted just before placing the hand in the water. Thirty seconds after hand immersion, participants were asked to rate the cold pain intensity (0-100) from the immersed hand followed by the second PPT measurement, and were informed to keep their hand in the water bath for as long as tolerable up to 1 minute. After the removal of the hand, the final PPT measurement was taken. This temperature was chosen based on prior experience with middle-aged and older adults with KOA, where 12 °C was found to produce moderate yet tolerable pain for most participants. Water was continually circulated and maintained at a constant temperature by a refrigeration unit (Neslab). An increase in PPT following cold water immersion demonstrated pain inhibition.

#### Pain Catastrophizing

The Coping Strategies Questionnaire-Revised measures the use of strategies for coping with pain by assessing 6 domains––distraction, catastrophizing, ignoring pain sensations, distancing from pain, coping self-statements, and praying. Participants rate how often they use specific strategies on a 7-point Likert scale from 0=never to 6=always, with higher scores indicating greater usage for each domain. This study used the 6-item catastrophizing subscale, with scores calculated as the mean of the responses. The reliability and validity of the Coping Strategies Questionnaire-Revised subscales have previously been shown to be acceptable [32,33].

#### Negative Affect

The Positive and Negative Affect Schedule includes 20 items that evaluate the frequency of both pleasant and unpleasant emotions individuals experience [31]. The inventory is divided into 2 subscales, each with 10 items for positive and negative emotions. Negative affect is calculated from the sum of 10 items (afraid, ashamed, distressed, guilty, hostile, irritable, jittery, nervous, scared, and upset), rated on a 5-point scale from 1=very slightly or not at all to 5=extremely. A lower total negative score indicates less negative affect (range: 10-50). The Positive and Negative Affect Schedule has been validated and demonstrates reliability, with an α coefficient range of .84 to .87 for negative affect [34].

### Statistical Analyses

Descriptive statistics were used to characterize the study participants. Chi-square or Fisher exact test for categorical variables and the 2-tailed *t* test for continuous variables were used to compare participant characteristics between the groups. Composite measures for QST were created by calculating *z* scores for the heat pain threshold and tolerance at the arm and knee; PPT at the medial and lateral aspects of the index knee, ipsilateral quadriceps, and trapezius; and PMP at the index patella and hand. The *z* scores for each pain measure were subsequently averaged across the body sites to yield overall heat pain threshold, heat pain tolerance, PPT, and PMP values for the analyses.

Separate path analytical models were estimated to assess the indirect effects (mediation) of ethnicity (coded 0 for non-Hispanic White and 1 for Asian American individuals) via pain catastrophizing or negative affect on each clinical and experimental pain measure. The path models facilitated the examination of both direct and indirect effects. Model fit, path coefficient estimates, and 95% highest posterior density CIs (“credibility” in Bayesian terms) for parameter estimates were generated using the Bayesian estimation method in Mplus (version 8.8; Muthén & Muthén). Bayesian estimation is advantageous in that it precludes the necessity of the normality assumption in the sampling distribution of estimates and potentially provides more accurate parameters in small-sample cases [35]. Model fit was evaluated using the criteria and methods recommended by Muthén and Asparouhov [36]. Where 95% CIs did not overlap with zero, the effect was considered significant.

### Ethical Considerations

The institutional review board (IRB) of the University of Arizona (UA) considers investigators engaged in research if they (1) interact with participants for research purposes, (2) have access to identifying study information, (3) obtain informed consent from research participants, or (4) the UA directly receives part of federal funds for the study (ie, UA is the prime awardee). If none of the earlier are true, then the researchers would not need IRB approval. Thus, this secondary analysis of deidentified data from an existing randomized controlled trial does not need any IRB approval. The original study (NCT04016272) received appropriate ethical approval, and written informed consent was obtained.

## Results

Table 1 presents the characteristics of the participants by race. The groups differed in terms of age (*P*=.001), BMI (*P*=.001), and Kellgren-Lawrence radiographic grade (*P*=.01). The mean age of non-Hispanic White individuals was 65.1 (SD 7.05) years, whereas the mean age of Asian American individuals was 54.8 (SD 7.36) years. The BMI for non-Hispanic White and Asian American individuals was 28.0 (SD 3.12) kg/m² and 25.0 kg/m² (SD 3.41) kg/m², respectively. Out of 20 Asian American individuals, most (n=11, 55%) were classified as grade 0. In contrast, grades 3 and 4 were predominant among non-Hispanic White individuals, with 7 out of 20 (35%) participants falling into these categories. Additionally, grade 2 was more common among non-Hispanic White individuals (8/20, 40%) compared with Asian American individuals (2/20, 10%). There were no significant differences between the groups in sex proportion, employment status, marital status, educational attainment, and household income.

**Table 1:** Basic characteristics of the participants (N=40)a.

Characteristic		Non-Hispanic White (n=20)	Asian American (n=20)	*P* value	
Age (years), mean (SD)		65.1 (7.05)	54.8 (7.36)	*.001*	
**Sex, n (%)**		8 (40)	13 (65)	.21	
	Male	12 (60)	7 (35)		
	Female	8 (40)	13 (65)		
BMI (kg/m^2^), mean (SD)		28.0 (3.12)	25.0 (3.41)	*.001*	
**Kellgren-Lawrence radiographic grade, n (%)**					*.010* ^b^
	0	2 (10)	11 (55)		
	1	3 (15)	5 (25)		
	2	8 (40)	2 (10)		
	3	6 (30)	2 (10)		
	4	1 (5)	0 (0)		
**Employment status, n (%)**					.28
	Yes	9 (47)	12 (71)		
	No	10 (53)	5 (29)		
**Marital status, n (%)**					.13^b^
	Married or partnered	13 (65)	18 (90)		
	Nonmarried or unpartnered	7 (35)	2 (10)		
**Educational attainment, n (%)**					.50
	2-year college degree or less	8 (40)	5 (25)		
	4-year college degree of higher	12 (60)	15 (75)		
**Household income (US $), n (%)**					.52
	More than 50,000	11 (58)	8 (42)		
	50,000 or less	8 (42)	11 (58)		

^a^Significant results are indicated in italics.

^b^Fischer exact test.

**Figure 1:**
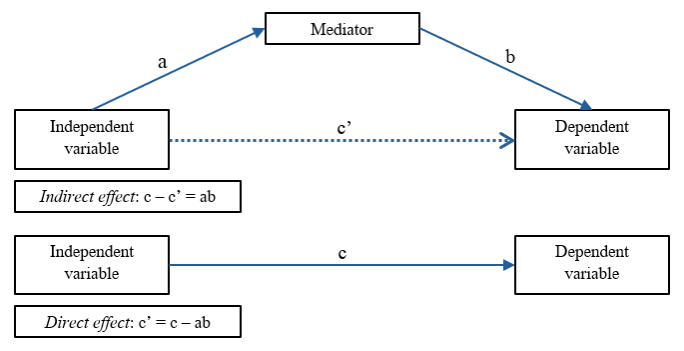
Mediation path model. For clinical pain measures, we measured WOMAC pain and SF-MPQ-2 pain. To produce composite QST measures, average z scores were computed for heat pain threshold and heat pain tolerance measurements at the arm and knee; PPT measurements at the medial and lateral aspect of the index knee, ipsilateral quadriceps, and trapezius; and PMP measurements at the patella and hand. a = direct effect of race on the mediator; b = direct effect of the mediator on pain measures after controlling for race; ab = indirect effect of race on pain measures operating through the mediator; c' = direct effect of race on pain measures after controlling for the mediator; c = total effect of race on pain measures without accounting for the mediator. Due to the small sample size, we analyzed each mediating effect (ie, pain catastrophizing and negative affect) separately. PMP: punctate mechanical pain; PPT: pressure pain threshold; QST: quantitative sensory testing; SF-MPQ-2: Short-Form McGill Pain Questionnaire-2; WOMAC: Western Ontario and McMaster Universities Osteoarthritis Index.

Descriptive statistics for variables used in the path models are presented in Table 2. Figure 1 shows the mediation path (race → mediator → pain). Fit for each of the models was acceptable, with all 95% CIs for the difference between observed and replicated chi-square values encompassing 0, all posterior predictive values >.45, and convergence of posterior parameter trace plots. Tables 3 and 4 provide results of the path analysis, including direct and indirect effects and the 95% highest posterior density CIs for each of the pain measure models.

**Table 2:** Descriptive statistics for pain-related outcomes, pain catastrophizing, and negative affect among non-Hispanic White and Asian American individuals (N=40).

Pain measures	Non-Hispanic White (n=20)	Asian American (n=20)
WOMAC^a^ pain (range: 0-20), mean (SD)	4.90 (2.55)	4.40 (2.67)
SF-MPQ-2^b^ continuous pain (range: 0-10), mean (SD)	1.78 (1.94)	1.98 (1.37)
SF-MPQ-2 intermittent pain (range: 0-10), mean (SD)	1.28 (2.19)	1.47 (1.83)
SF-MPQ-2 neuropathic pain (range: 0-10), mean (SD)	0.67 (0.93)	1.07 (1.24)
SF-MPQ-2 affective pain (range: 0-10), mean (SD)	0.79 (1.83)	1.46 (1.66)
Heat pain threshold^c^, mean (SD)	0.49 (0.82)	–0.49 (0.74)
Heat pain tolerance^c^, mean (SD)	0.51 (0.76)	–0.51 (0.73)
Pressure pain threshold^c^, mean (SD)	0.45 (0.86)	–0.45 (0.61)
Punctate mechanical pain^c^, mean (SD)	–0.62 (0.56)	0.62 (0.76)
Conditioned pain modulation, mean (SD)	1.29 (1.14)	1.16 (0.71)
Pain catastrophizing (range: 0-6), mean (SD)	0.31 (0.74)	1.33 (1.25)
Negative affect (range: 10-50), mean (SD)	14.00 (4.29)	20.15 (9.02)

^a^WOMAC: Western Ontario and McMaster Universities Osteoarthritis Index.

^b^SF-MPQ-2: Short-Form McGill Pain Questionnaire-2.

^c^Average *z* score.

In each model (Table 3), the direct effect of race on pain catastrophizing (Figure 1; a = direct effect of race on the mediator) indicated that Asian American individuals had significantly higher pain catastrophizing scores than non-Hispanic White individuals (mean difference 1.03; 95% CI 0.29-1.70). Additionally, as shown in Table 4, in each model, the direct effect of race on negative affect indicated that Asian American individuals yielded significantly higher negative affect scores than non-Hispanic White individuals (mean difference 6.15; 95% CI 1.11-10.80).

**Table 3:** Estimated direct and indirect effects with 95% CI (N=40; pain catastrophizing as a mediator)^a,b^.

Pain outcomes (*R^2^*)	Direct effects		Indirect effect, ab (95% CI)
	c’ (95% CI)	b (95% CI)	
WOMAC^c^ pain (0.179)	–1.53 (–3.39 to 0.25)	1.00 (0.21-1.81)	0.96 (0.03-2.16)
SF-MPQ-2^d^ continuous pain (0.283)	–0.67 (–1.78 to 0.40)	0.85 (0.38-1.34)	0.84 (0.18-1.70)
SF-MPQ-2 intermittent pain (0.187)	–0.64 (–2.07-0.73)	0.81 (0.20-1.43)	0.78 (0.03-1.71)
SF-MPQ-2 neuropathic pain (0.215)	–0.06 (–0.83 to 0.67)	0.45 (0.12-0.78)	0.43 (0.03-0.95)
SF-MPQ-2 affective pain (0.410)	–0.41 (–1.47 to 0.60)	1.06 (0.61-1.52)	1.05 (0.24-1.99)
Heat pain threshold^e^ (0.284)	–1.07 (–1.67 to –0.50)	0.08 (–0.17 to 0.34)	0.07 (–0.20 to 0.39)
Heat pain tolerance^e^ (0.304)	–0.94 (–1.52 to –0.39)	–0.07 (–0.32 to 0.18)	–0.06 (–0.38 to 0.19)
Pressure pain threshold^e^ (0.259)	–0.96 (–1.54 to –0.41)	0.06 (–0.19 to 0.31)	0.05 (–0.23 to 0.34)
Punctate mechanical pain^e^ (0.462)	1.09 (0.58-1.57)	0.15 (–0.07 to 0.37)	0.14 (–0.09 to 0.43)
Conditioned pain modulation (0.168)	–0.49 (–1.70 to 0.16)	0.36 (0.07-0.65)	0.34 (0.00-0.79)

^a^Significant results are indicated in italics.

^b^c’ = direct effect of race on pain measures after controlling for pain catastrophizing; b = direct effect of pain catastrophizing on pain measures after controlling for race; ab = indirect effect of race on pain measures operating through pain catastrophizing.

^c^WOMAC: Western Ontario and McMaster Universities Osteoarthritis Index.

^d^SF-MPQ-2: Short-Form McGill Pain Questionnaire-2.

^e^Average *z* score.

**Table 4:** Estimated direct and indirect effects with 95% CI (N=40; negative affect as a mediator)^a,b^.

Pain outcomes (*R^2^*)	Direct effects		Indirect effect, ab (95% CI)
	c’ (95% CI)	b (95% CI)	
WOMAC^c^ pain (0.091)	–1.05 (–2.92 to 0.83)	0.09 (–0.03 to 0.22)	0.50 (–0.23 to 1.59)
SF-MPQ-2^d^ continuous pain (0.094)	–0.17 (–1.36 to 1.04)	0.06 (–0.02 to 0.14)	0.34 (–0.13 to 1.04)
SF-MPQ-2 intermittent pain (0.155)	–0.45 (–1.83 to 0.95)	0.11 (0.02-0.20)	0.59 (–0.04 to 1.48)
SF-MPQ-2 neuropathic pain (0.186)	0.04 (–0.70 to 0.80)	0.06 (0.01-0.11)	0.34 (–0.03 to 0.80)
SF-MPQ-2 affective pain (0.108)	0.32 (–0.93 to 1.59)	0.06 (–0.02 to 0.14)	0.32 (–0.17 to 1.04)
Heat pain threshold^e^ (0.274)	–1.01 (–1.58 to –0.43)	0.00 (–0.03 to 0.04)	0.02 (–0.25 to 0.27)
Heat pain tolerance^e^ (0.297)	–1.01 (–1.56 to –0.45)	–0.00 (–0.04 to 0.04)	–0.00 (–0.25 to 0.26)
Pressure pain threshold^e^ (0.255)	–0.94 (–1.49 to –0.38)	0.01 (–0.03 to 0.04)	0.03 (–0.22 to 0.29)
Punctate mechanical pain^e^ (0.453)	1.13 (0.65-1.62)	0.02 (–0.01 to 0.05)	0.09 (–0.10 to 0.36)
Conditioned pain modulation (0.039)	–0.15 (–0.85 to 0.56)	0.01 (–0.04 to 0.05)	0.02 (–0.31 to 0.33)

^a^Significant results are indicated in italics.

^b^c’ = direct effect of race on pain measures after controlling for pain catastrophizing; b = direct effect of pain catastrophizing on pain measures after controlling for race; ab = indirect effect of race on pain measures operating through pain catastrophizing.

^c^WOMAC: Western Ontario and McMaster Universities Osteoarthritis Index.

^d^SF-MPQ-2: Short-Form McGill Pain Questionnaire-2.

^e^Average *z* score.

### Direct Effect of Race on Pain Measures After Controlling Pain Catastrophizing

The direct effect of race on the heat pain threshold (c’=–1.07), heat pain tolerance (c’=–0.94), and PPT (c’=–0.96) indicated that Asian American individuals had lower mean scores on these experimental pain measures than non-Hispanic White individuals after controlling for pain catastrophizing. The direct effect of race on PMP (c’=1.09) indicated that Asian American individuals generated higher mean scores on this pain measure than non-Hispanic White individuals after controlling for pain catastrophizing. After controlling for pain catastrophizing, Asian American and non-Hispanic White participants yielded similar mean scores for clinical pain measures.

### Direct and Indirect Effect of Pain Catastrophizing on Pain Measures

Pain catastrophizing exhibited positive direct effects, while race exerted positive indirect effects through pain catastrophizing on WOMAC (b=1.00, ab=0.96), SF-MPQ-2 continuous (b=0.85, ab=0.84), SF-MPQ-2 intermittent (b=0.81, ab=0.78), SF-MPQ-2 neuropathic (b=0.45, ab=0.43), and SF-MPQ-2 affective (b=1.06, ab=1.05) pain. These results indicate that participants with higher pain catastrophizing scores tend to have higher values on these pain measures after controlling for race. Considering that race had no direct effects on these pain scores but exerted indirect effects via pain catastrophizing, the effects of race were likely fully mediated through pain catastrophizing for these measures. After controlling race, we did not identify any direct effects of pain catastrophizing on the heat pain threshold, heat pain tolerance, PPT, and PMP. Surprisingly, pain catastrophizing exhibited positive direct effects on CPM (b=0.36).

### Direct Effect of Race on Pain Measures After Controlling for Negative Affect

Based on the direct effect of race on the heat pain threshold (c’=–1.01), heat pain tolerance (c’=–1.01), and PPT (c’=–0.91), Asian American individuals yielded lower mean scores on these experimental pain sensitivity measures than non-Hispanic White individuals after controlling for negative affect. The direct effect of race on PMP (c’= 1.13) indicated that Asian American individuals had higher mean scores on this pain measure than non-Hispanic White individuals after controlling for negative affect. Race had no direct effect on CPM. After controlling for negative affect, Asian American and non-Hispanic White participants were found to have similar mean scores for all clinical pain measures.

### Direct and Indirect Effects of Negative Affect on Pain Measures

We exclusively detected direct effects of negative affect on SF-MPQ-2 intermittent (b=0.11) and SF-MPQ-2 neuropathic (b=0.06) pain after controlling for race. Race exerted no indirect effects on pain measures via negative affect in any of the path models.

## Discussion

### Principal Findings

This study investigated whether variations in pain catastrophizing and negative affect explain group differences in clinical and experimental pain between non-Hispanic White and Asian American individuals with KOA. Our main finding suggests that Asian American individuals show higher levels of pain catastrophizing than non-Hispanic White individuals and that it plays a relevant role in greater clinical pain in Asian American individuals. The results additionally indicate that participants with higher pain catastrophizing scores tended to have higher WOMAC- and SF-MPQ-2-measured clinical pain. Furthermore, Asian American individuals exhibited greater sensitivity to experimental pain compared with non-Hispanic White individuals; however, this difference was not due to pain catastrophizing. Asian American individuals also reported experiencing more negative affect than non-Hispanic White individuals, yet this did not seemingly influence the relationship between race and any pain measures.

### Comparison With Prior Work

Disparities in pain catastrophizing based on race and ethnicity have been documented in individuals with KOA [13,37-39] and various other pain-related medical conditions [40-43]; notably, individuals who self-identify as minorities, including Black and Hispanic or Latinx groups, are reported to engage in pain catastrophizing as a pain coping strategy more frequently than their non-Hispanic White peers. Increasing evidence also suggests that catastrophizing is a significant mediator of race differences in clinical pain. A recent study by Fullwood et al [39] found that pain catastrophizing mediated the relationship between race (Black vs non-Hispanic White individuals) and WOMAC pain in adults with KOA. Similar findings were reported in the study by Lane et al [43] on individuals with chronic spinal pain receiving physical therapy and the study by Fabian et al [44] in healthy pain-free samples. Our study expands on previous investigations by identifying pain catastrophizing as a significant contributor to group differences in clinical pain between non-Hispanic White and Asian American individuals.

Extensive prior research has demonstrated that pain catastrophizing is associated with undesirable pain outcomes, including more frequent pain experiences or greater pain intensity [11-13]. However, our finding that Asian American individuals have higher clinical pain scores with increasing levels of pain catastrophizing represents a novel contribution to the field. The exact reasons underlying the significantly higher pain catastrophizing scores among Asian American individuals compared with non-Hispanic White individuals remain unclear. A possible explanation is the relationship between acculturative stress and pain catastrophizing in Asian American individuals. Ahn et al [14] suggest that chronic stress contributes to increased pain perception, potentially owing to its physical impact from chronically high levels of sympathetic activation and subsequent physiologic exhaustion; this, in turn, may reduce one’s ability to cope with the added stress of pain [45]. They further note that a major source of chronic stress for immigrants could be the process of acculturation [46,47]. In addition, higher pain catastrophizing scores among Asian American individuals could be attributed to a cultural emphasis on pain-related stoicism in Asian communities, which may discourage openly expressing chronic pain to avoid burdening others [48,49]. This cultural disposition causes Asian American individuals to suffer silently, thus amplifying their mental agony. Furthermore, Asian cultural communication styles often prioritize indirectness and subtlety, which can result in less effective communication with health care providers from different cultural backgrounds regarding pain experiences [50]. This communication gap may hinder effective pain management and perpetuate a cycle of unexpressed and poorly managed KOA pain, thereby contributing to higher levels of pain catastrophizing compared to non-Hispanic White individuals. Furthermore, immigrants’ experiences with the health care system and the challenges they encounter in accessing adequate pain treatment may lead to poorer pain outcomes. This may foster negative thinking about their pain and may leave Asian American patients feeling that their pain is unmanageable and will inevitably worsen.

Asian American individuals reported greater experimental pain sensitivity (heat pain, PPT, and PMP) than non-Hispanic White individuals, replicating previous findings on middle-aged and older adults with KOA [9] and similar reports on younger Asian American individuals [51]. Furthermore, heightened sensitivity occurred at both the affected knee and unaffected body sites, suggesting increased central sensitization. However, pain catastrophizing could not explain the racial group differences in any measures of experimental pain in this study. This contradicts previous studies wherein pain catastrophizing was found to influence racial group differences in QST measures among nonclinical samples [52-55] and patients with chronic low back pain [41]. Several explanations can be proposed for such findings. First, Meints et al [55] found that racial group differences in cold pain tolerance (non-Hispanic White vs African American individuals) were mediated by the rumination component of pain catastrophizing but not by the magnification or helplessness components, examining the mediatory effects of different pain catastrophizing components may yield varied results. Second, other critical factors, such as biological, genetic, social, and environmental mechanisms, may also influence the observed differences. For instance, Rowell et al [51] found that differences in endogenous pain regulatory mechanisms, such as mean arterial pressure and heart rate, potentially play a role in the differences in experimental pain sensitivity between young non-Hispanic White and Asian American individuals. Based on earlier evidence, genetic links to pain phenotypes differ according to racial or ethnic group, potentially generating dissimilarities in pain sensation. For example, pain sensitivity has been associated with variations in the catechol-O-methyltransferase [56] and μ-opioid receptor genes [57]. Moreover, frequency differences in the alleles of pain-related gene polymorphisms may contribute to racial and ethnic disparities in pain responses [58]. Furthermore, studies have suggested a role for nutritional supplement status [59], lower sociodemographic resources [60], and racial discrimination [61] in accounting for individual or racial and ethnic differences in experimental pain sensitivity—all of which potentially contributed to the observed differences but require further evaluation in the future.

Asian American individuals had significantly higher negative affect scores than non-Hispanic White individuals. Although higher negative affect scores were strongly correlated with both intermittent and neuropathic pain (measured using the SF-MPQ-2), negative affect did not seem to influence the relationship between race and any pain measures. Various negative affect-related constructs are important to pain; nevertheless, they differ in specificity and are conceptually distinct; some constructs are general, such as anxiety, depression, and negative affect, whereas others are more specifically pain-related, such as fear of pain, pain anxiety, and pain catastrophizing [62]. Overall, our preliminary findings suggest that pain-specific variables (ie, pain catastrophizing) should be prioritized over general negative affect to minimize pain disparities between non-Hispanic White and Asian American individuals. However, further studies involving larger sample sizes are necessary to confirm our findings. In fact, a study by Ahn et al [9], which established that higher depression levels in Asian American than in non-Hispanic White individuals explained racial group differences in clinical and experimental pain, included 50 participants per group. Additionally, we could not account for covariates, such as sex, age, and pain-related medication, owing to the small sample sizes in the path models, which might have affected the results.

### Strengths and Limitations

Our study is the first to highlight the crucial role of pain catastrophizing in explaining disparities in clinical KOA pain between non-Hispanic White and Asian American individuals, contributing to the growing body of literature on racial group differences in pain among individuals with KOA and its associated psychological conditions. Further, the study’s strength was upheld by its comprehensive examination of pain in Asian American individuals using a wide range of pain measures, focusing on a population that has received limited attention in studies assessing and managing KOA pain.

This study has certain limitations. First, the findings may not be generalizable as they are based on a convenience sample from a specific region. Moreover, the Asian American participants in the study were limited to English speakers. These limitations introduce challenges in interpreting the findings, underscoring the need for samples from other regions and a more diverse group of Asian American individuals for cross-validation. Second, as previously mentioned, a key limitation of this study is the lack of information about potential commonalities or differences within our broadly categorized, monolithic Asian American sample. Therefore, caution is warranted when interpreting our conclusions. Furthermore, although we use the term “Asian American” when referring to the prior works, we acknowledge the significant heterogeneity within this population, including the diverse countries of origin of participants in individual studies and the considerable variation across “Asian American” cohorts in different studies. Thus, our discussion on racial and ethnic differences should be carefully interpreted. Third, this pilot study had a small sample size. Consequently, statistical analyses were constrained, and data outliers were more likely to skew the results, highlighting the need for a larger sample size. Furthermore, the combination of the small sample size and the lack of subgroup information on the Asian American sample limited the study’s capacity to be specifically designed or sufficiently powered to explore variations within smaller subgroups of Asian American individuals. Fourth, the cross-sectional design hindered our ability to discern the directionality of the relationships between variables. Indeed, evidence suggests that pain catastrophizing may not be a characterological trait but a complex phenomenon that can both affect and be affected by pain [39]. Kim et al [48] argue that as clinical pain scores increase, a sense of helplessness or an inability to control chronic knee pain may develop, contributing to higher levels of pain catastrophizing in Asian American individuals. These important relationships warrant further investigation in future studies. Furthermore, previous studies have evaluated pain catastrophizing over a longitudinal period to better understand its influence on pain over time in adults with KOA [39]. Similar studies should also be conducted in Asian American samples. Finally, we acknowledge that the term “pain catastrophizing” can considered pejorative and stigmatizing, conflicting with patient-centered care approaches [63]. Labeling patients in this manner potentially leads to blame and stereotyping, adversely affecting decision-making and care quality. Recent analyses have proposed that “pain-related worrying” and “pain-related distress” may better capture the essence of what is measured by pain-catastrophizing items [64].

### Future Directions

This pilot study’s findings provide a crucial foundation for future research and clinical practice. Considering the limited sample size, we analyzed each mediatory effect separately. Future studies including larger samples may use more sophisticated models to concurrently examine a broader range of factors, thereby more comprehensively elucidating the mechanisms underlying racial disparities in pain between non-Hispanic White and Asian American individuals; in addition to pain catastrophizing and negative affect, as previously discussed, future research should investigate biological, genetic, and other psychological variables essential to understanding chronic KOA pain and evaluate them as explanatory mechanisms to develop more tailored interventions.

Additionally, it is important to acknowledge that the lumping of Asian American groups together in the current study is problematic, as it obscures the tremendous diversity and complexity within and across these groups. This approach may have excluded individuals with varying levels of pain catastrophizing and negative affect or overlooked how acculturative stress and cultural practices—factors that can vary greatly between Asian American subgroups—may influence pain perception and response. Therefore, future studies should account for the demographic and social construction of the Asian American category and its implications in KOA pain research to ensure nuanced and culturally informed analyses.

Finally, our findings underscore the need to systematically assess and treat pain catastrophizing in Asian American individuals in health care settings to ensure effective pain management. Interventions targeting this maladaptive cognitive style among Asian American individuals may help mitigate racial disparities in clinical pain. In particular, the interventions should be culturally sensitive and tailored by further scrutinizing the factors influencing pain catastrophizing in Asian American individuals. If acculturative stress influences pain catastrophizing, then therapy for pain catastrophizing (eg, cognitive behavioral therapy) could be enhanced by focusing on culturally sensitive stress management techniques. In addition, a better understanding of differences in pain experiences based on race, sociocultural background, and experiences with the health care system—such as Asian communities facing structural and systemic barriers that influence pain—may help reduce disparities in pain management.

### Conclusions

This pilot study examined psychological factors, specifically pain catastrophizing and negative affect, as potential explanatory mechanisms behind racial group differences in clinical and experimental pain between non-Hispanic White and Asian American individuals with KOA pain. Apparently, pain catastrophizing is essential to addressing racial disparities in clinical KOA pain; however, further research is warranted to verify our findings and elucidate unresolved mechanisms.

## Abbreviations

CPM: conditioned pain modulation
IRB: institutional review board
KOA: knee osteoarthritis
PMP: punctate mechanical pain
PPT: pressure pain threshold
QST: quantitative sensory testing
SF-MPQ-2: Short-form-McGill Pain Questionnaire-2
UA: University of Arizona
WOMAC: Western Ontario and McMaster Universities Osteoarthritis Index

## Data Availability

The datasets generated during and/or analyzed during this study are available from the corresponding author on reasonable request.
